# Supplementary material for: Systematic Analysis of Biological Processes Reveals Gene Co-expression Modules Driving Pathway Dysregulation in Alzheimer’s Disease
Source: Aging Dis. 2024 Jun 12;16(3):1598–625. doi: 10.14336/AD.2024.0429 (PMC12096932; doi:10.14336/AD.2024.0429)
Supplement: Supplementary file 1 — The Supplementary data can be found online at: www.aginganddisease.org/EN/10.14336/AD.2024.0429. [file AD-16-3-1598-s.pdf]

SUPPLEMENTARY DATA

Systematic Analysis of Biological Processes Reveals Gene Co-expression Modules Driving Pathway Dysregulation in Alzheimer's Disease

Temitope Adeoye, Syed I Shah, Ghanim Ullah

© 2024. Adeoye T et al. Published online at http://www.aginganddisease.org/EN/10.14336/AD.2024.0429

SUPPLEMENTARY DATA

Supplementary Tables

Supplementary Table 1. Sample metadata for all 20 donors, including post-mortem neuropathological assessments, clinical evaluations, and pathological grouping. **Supplementary Tables 2—4.** The table of p-values and log fold changes for all genes included in the differential analysis test across all brain regions (MTG 2; STG 3; ETC 4) and cell types. Supplementary Table 5. Table of pathway renaming conventions. **Supplementary Tables 6—8.** Comprehensive documentation of the pathway analysis results for each brain region (MTG 6; SFG 7; ETC 8) with detailed statistical results (coefficient estimates and p-values) for the prioritized candidate pathways identified across major cell types. **Supplementary Tables 9—11.** Results from DME analysis for each brain region (MTG 9; SFG 10; ETC 11) across all cell types. **Supplementary Table 12.** List of overlapping dysregulated pathways along with corresponding statistics for each cell type. **Supplementary Table 13.** List of hub genes and hDEGs in each module for all cell types across brain regions. Overlapping hub genes are presented in **Supplementary Table 14.** All supplementary materials are publicly available at: https://zenodo.org/records/11529902 [46].

Supplementary Figures

Supplementary Fig. 1. Principal Component Analysis (PCA) of aggregated pseudoreplicates highlighted with relevant metadata. PC2 and PC3 were chosen because other components do not distinctly show clustered cell types. **Supplementary Fig. 2.** Overlapping dysregulated biological processes across brain regions. **Supplementary Figs. 3—5.** Soft power thresholds demonstrating a fit to the scale-free topology model across all cell types in each brain region (MTG 3; SFG 4; ETC 5). **Supplementary Fig. 6—8.** Dendrograms showing the different co-expression modules resulting from the network analysis across cell types in each brain region (MTG 6; SFG 7; ETC 8).

© 2024. Adeoye T et al. Published online at http://www.aginganddisease.org/EN/10.14336/AD.2024.0429

SUPPLEMENTARY DATA

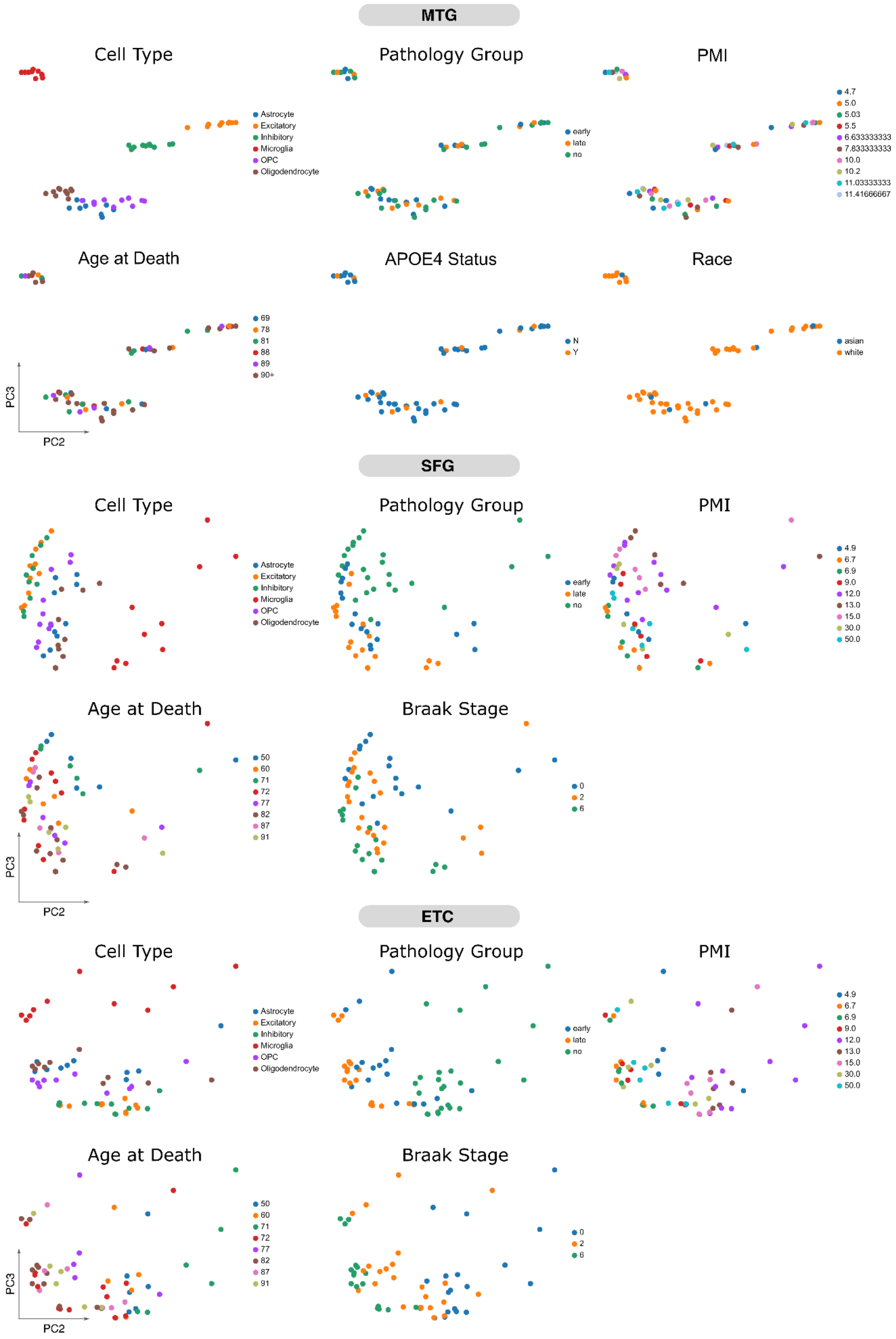

© 2024. Adeoye T et al. Published online at http://www.aginganddisease.org/EN/10.14336/AD.2024.0429

SUPPLEMENTARY DATA

Supplementary Figure 1. Principal Component Analysis (PCA) of Aggregated Pseudoreplicates Highlighted with Relevant Metadata. PCA plots of aggregated pseudoreplicates, with each point color-colored according to available metadata. Data from SFG, MTG, and ETC presented in the top, middle, and bottom panels respectively. Colour gradations within each plot segment correlate with metadata variations, illustrating potential confounding variables in expression data.

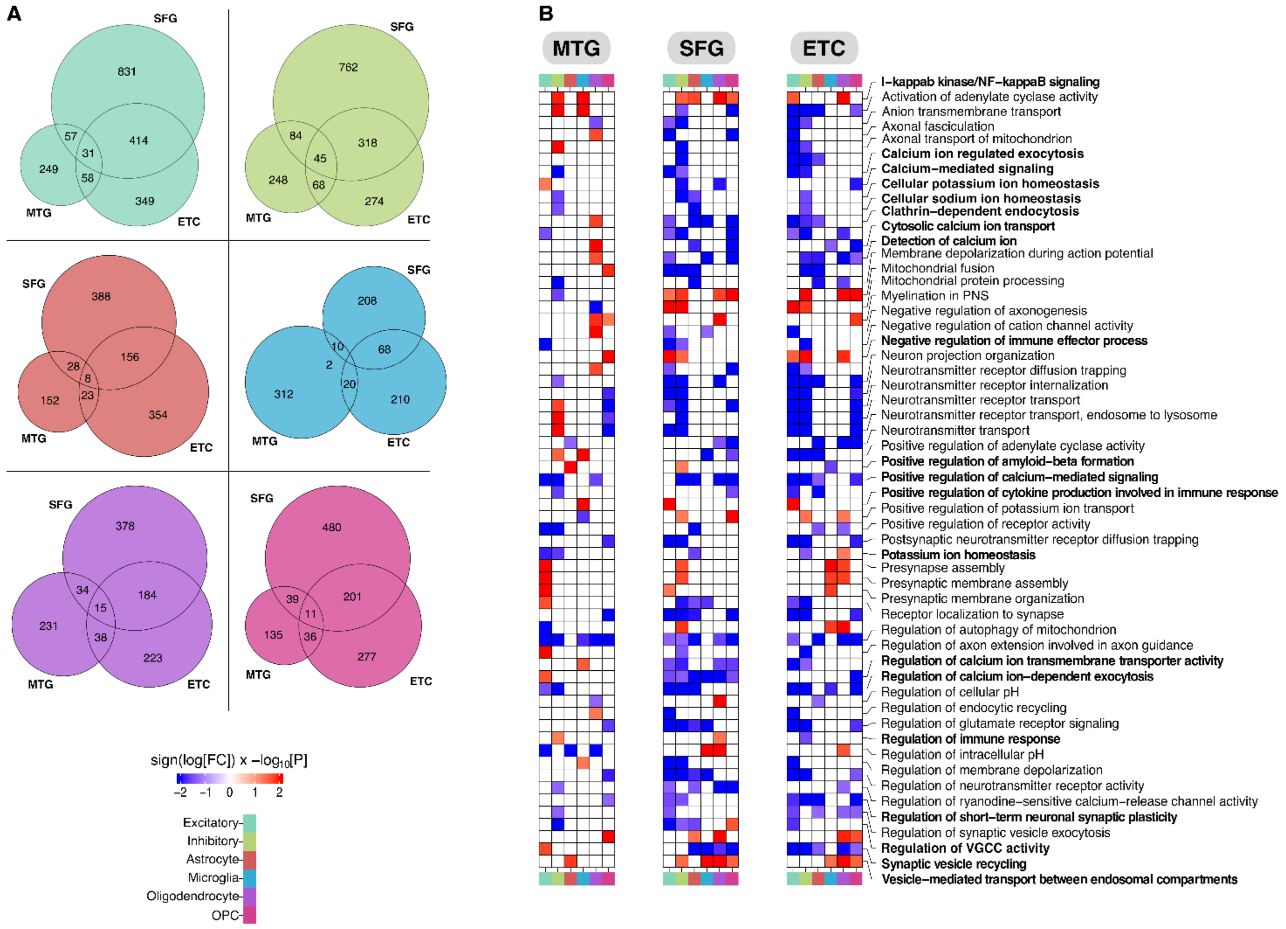

Supplementary Figure 2. Overlapping dysregulated biological processes across brain regions. (**A**), Venn diagram showing overlapping pathways across brain regions in each cell type. (**B**), Heatmaps depicting select overlapping Gene Ontology biological processes dysregulated in at least one cell type in each brain region (nominal $P < 0.05$). Red indicates upregulation while blue indicates downregulation. Pathways discussed in the Article are highlighted in bold text.

© 2024. Adeoye T et al. Published online at http://www.aginganddisease.org/EN/10.14336/AD.2024.0429

SUPPLEMENTARY DATA

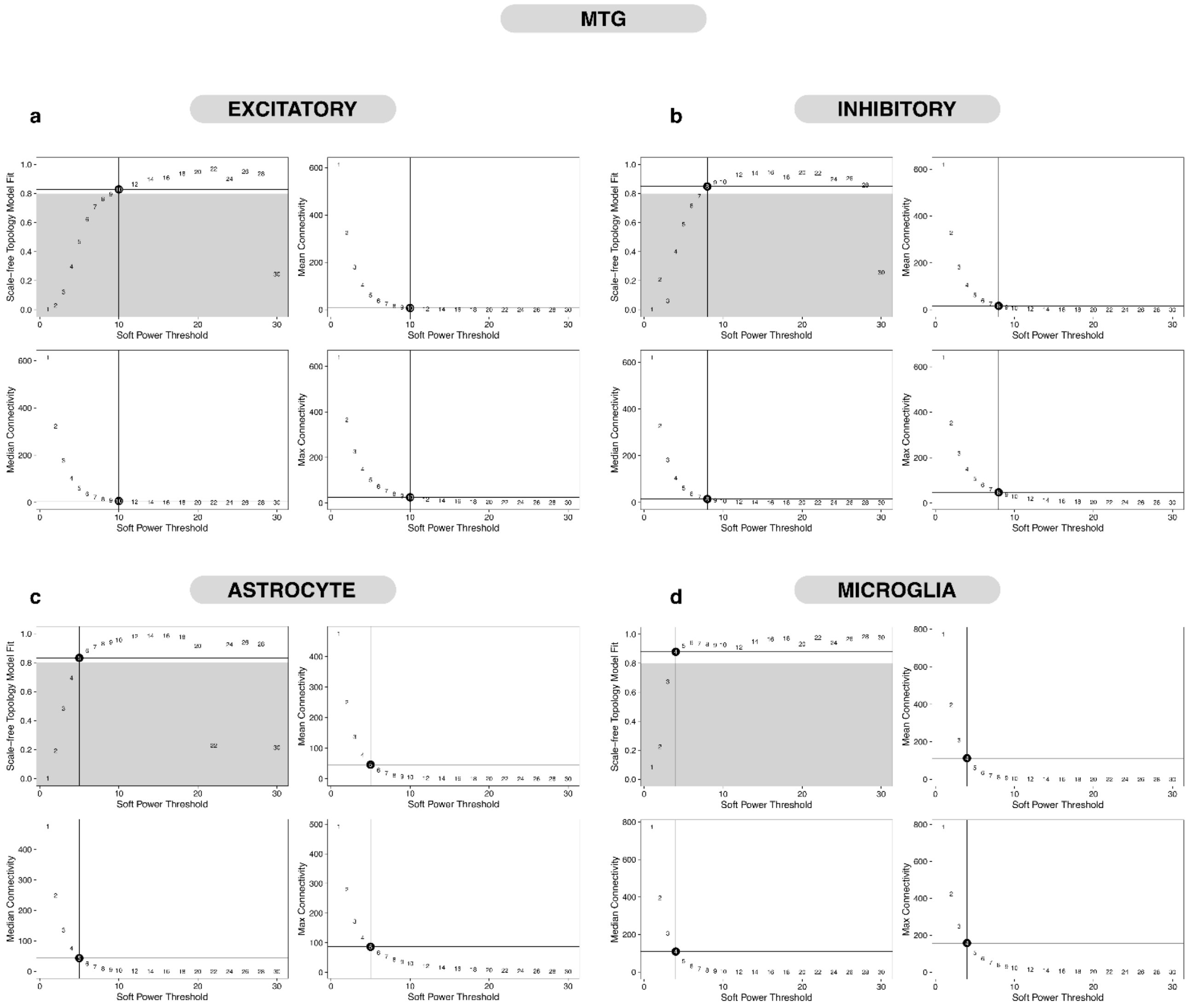

Supplementary Figure 3. Soft power thresholds demonstrating a fit to the scale-free topology model across all cell types in the MTG study. Each curve represents the relationship between the chosen soft-thresholding power and the scale-free topology model fit for a respective cell type for subsequent network analysis.

© 2024. Adeoye T et al. Published online at http://www.aginganddisease.org/EN/10.14336/AD.2024.0429

SUPPLEMENTARY DATA

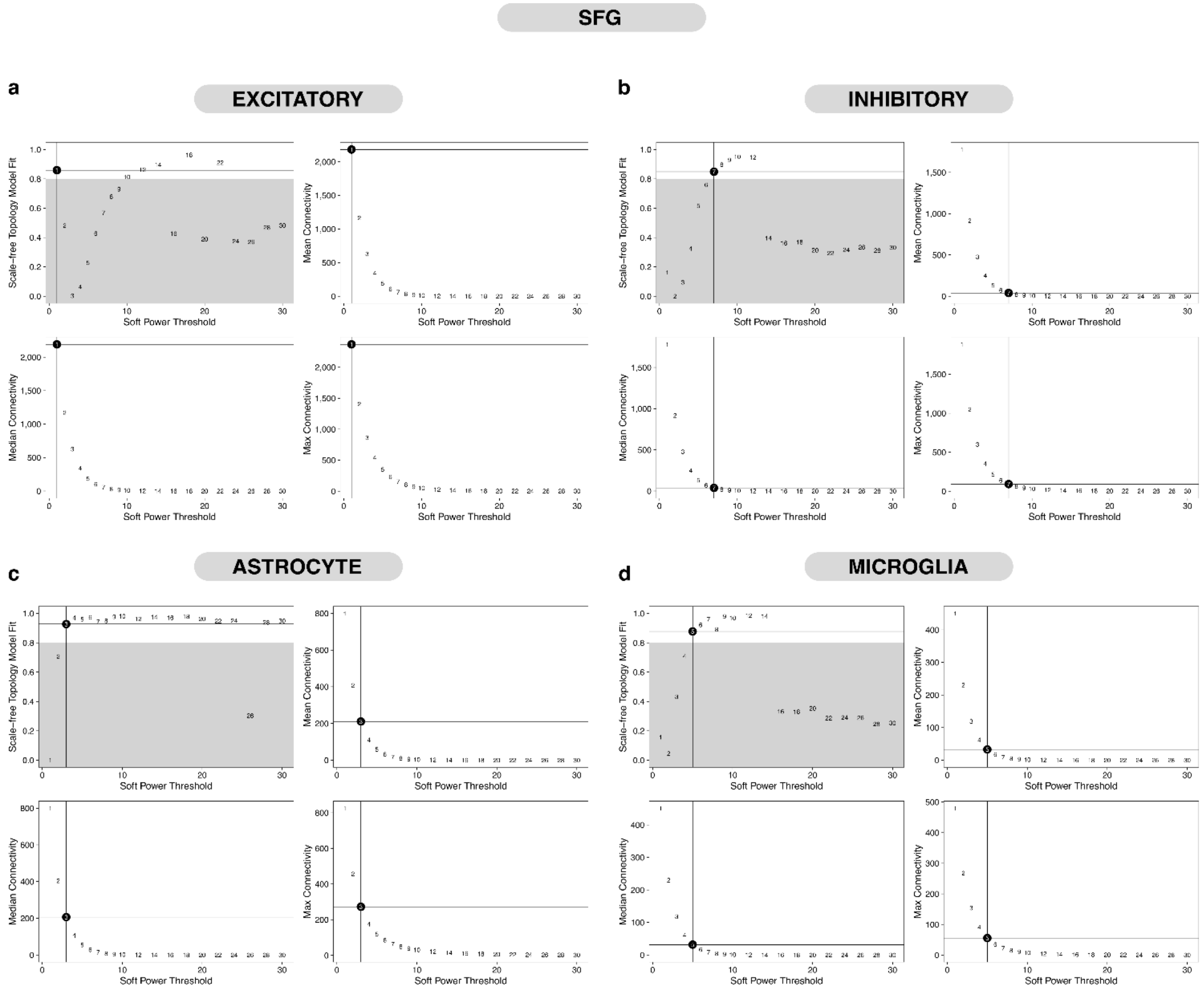

Supplementary Figure 4. Soft power thresholds demonstrating a fit to the scale-free topology model across all cell types in the SFG study. Each curve represents the relationship between the chosen soft-thresholding power and the scale-free topology model fit for a respective cell type for subsequent network analysis.

© 2024. Adeoye T et al. Published online at http://www.aginganddisease.org/EN/10.14336/AD.2024.0429

SUPPLEMENTARY DATA

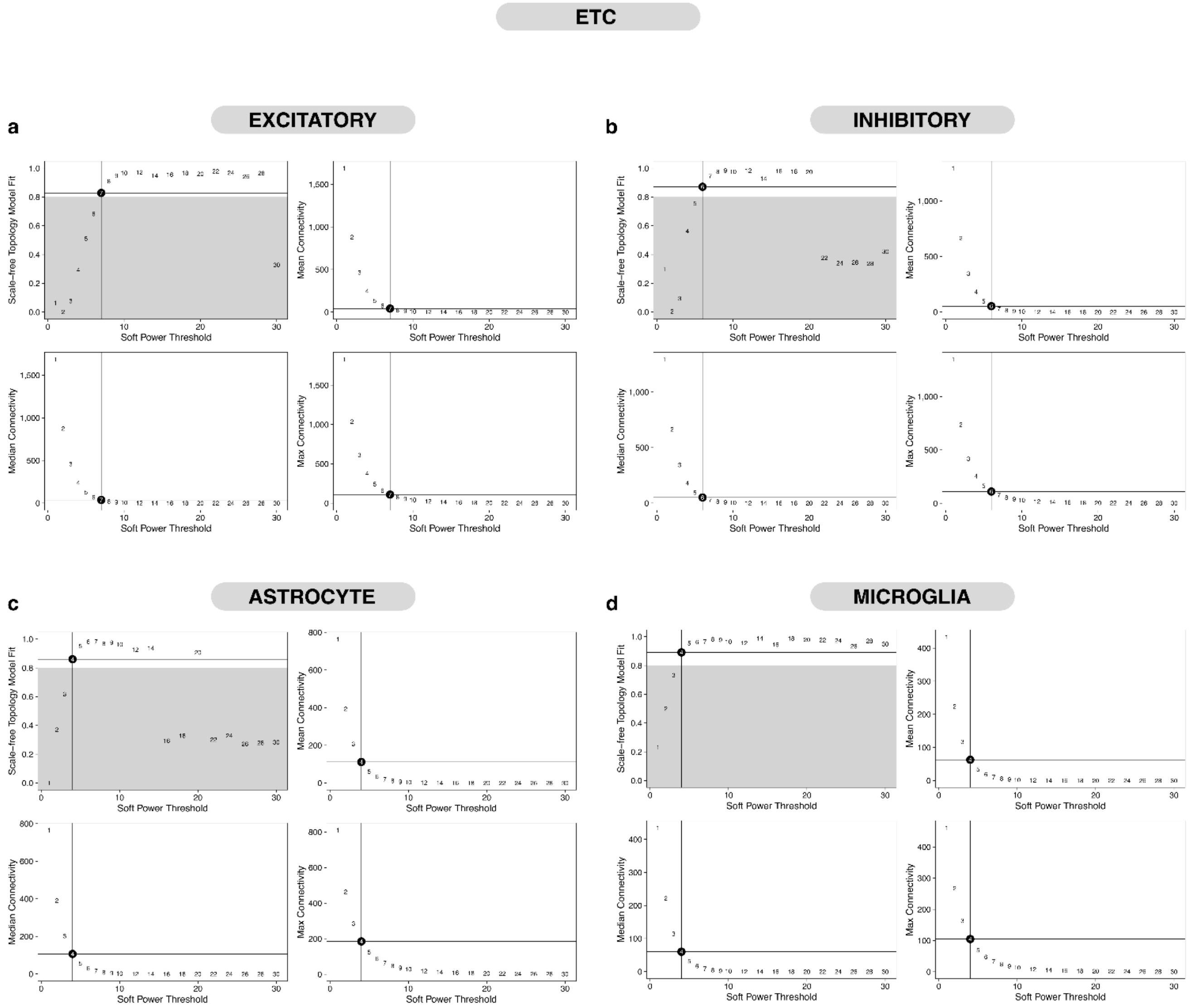

Supplementary Figure 5. Soft power thresholds demonstrating a fit to the scale-free topology model across all cell types in the ETC study. Each curve represents the relationship between the chosen soft-thresholding power and the scale-free topology model fit for a respective cell type for subsequent network analysis.

© 2024. Adeoye T et al. Published online at http://www.aginganddisease.org/EN/10.14336/AD.2024.0429

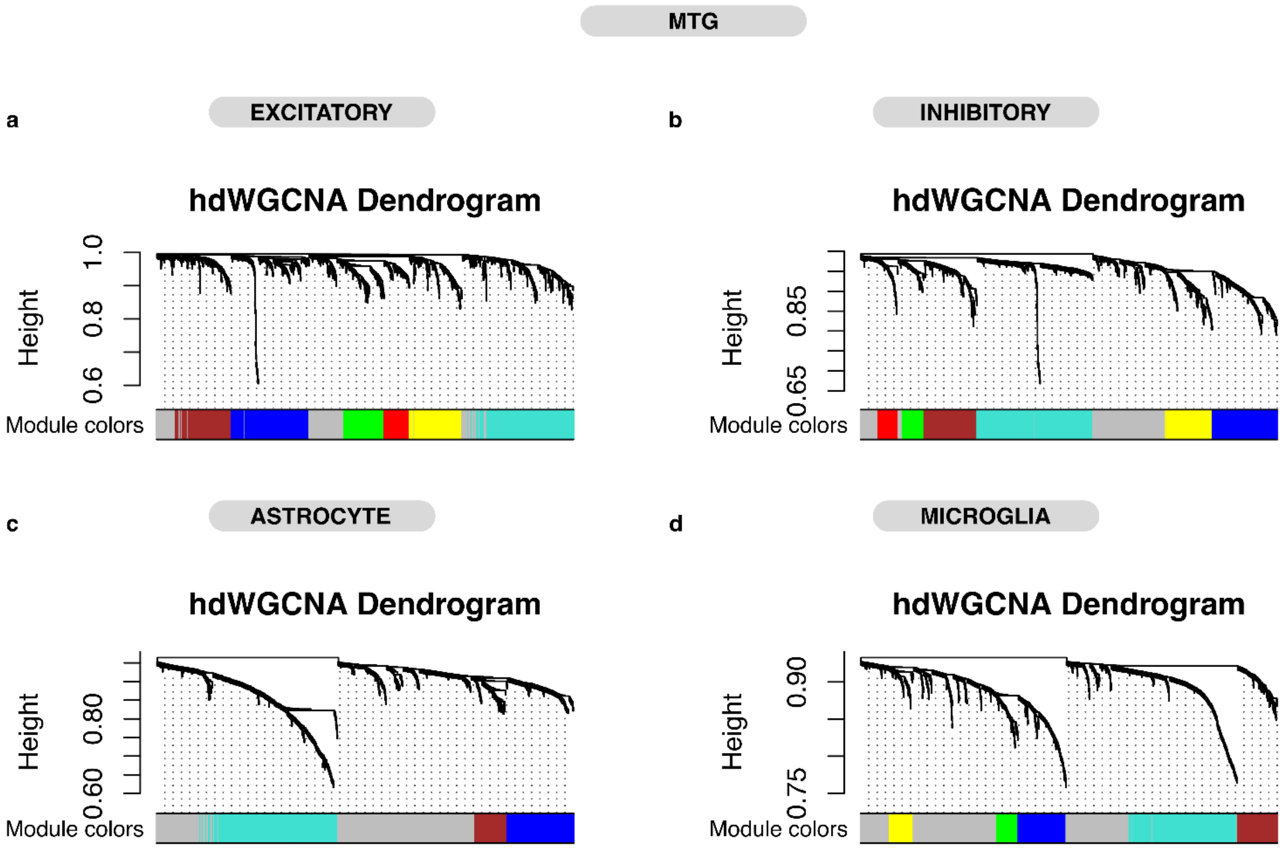

Supplementary Figure 6. Dendrograms showing the different co-expression modules resulting from the network analysis in the MTG. Each leaf of the dendrogram represents a single gene, while the module colours signify the corresponding co-expression module assignments.

© 2024. Adeoye T et al. Published online at http://www.aginganddisease.org/EN/10.14336/AD.2024.0429

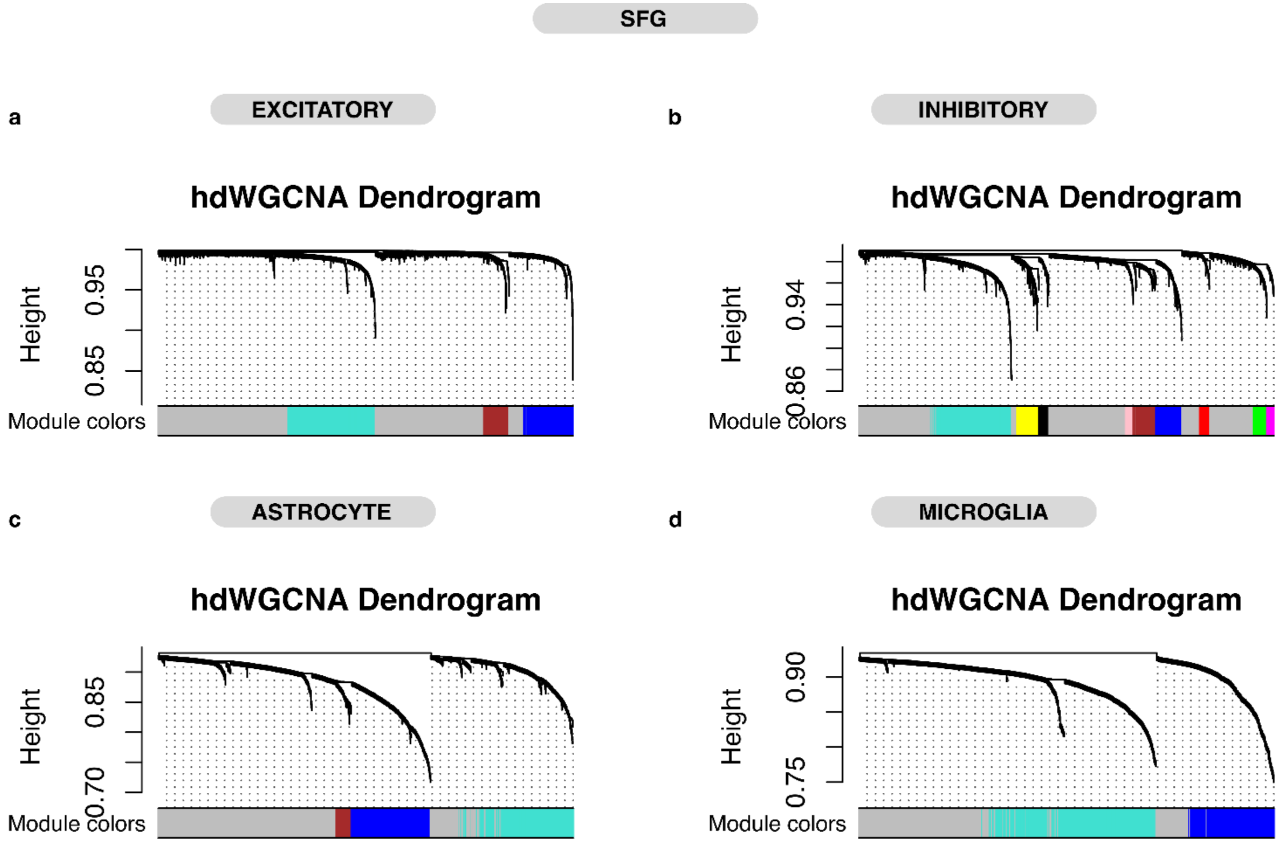

Supplementary Figure 7. Dendrograms showing the different co-expression modules resulting from the network analysis in the SFG. Each leaf of the dendrogram represents a single gene, while the module colours signify the corresponding co-expression module assignments.

© 2024. Adeoye T et al. Published online at http://www.aginganddisease.org/EN/10.14336/AD.2024.0429

SUPPLEMENTARY DATA

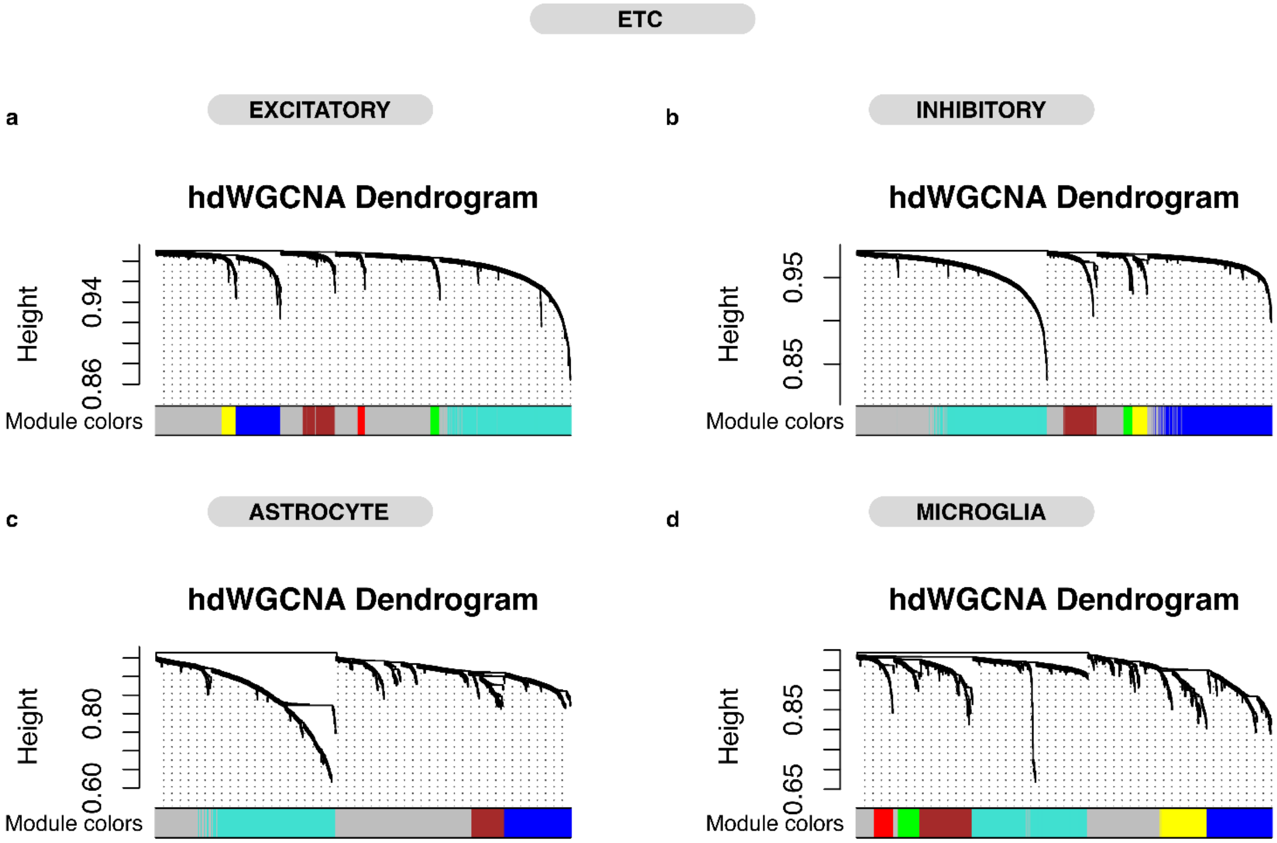

Supplementary Figure 8. Dendrograms showing the different co-expression modules resulting from the network analysis in the ETC. Each leaf of the dendrogram represents a single gene, while the module colours signify the corresponding co-exp expression module assignments.

© 2024. Adeoye T et al. Published online at http://www.aginganddisease.org/EN/10.14336/AD.2024.0429
